# Rupture discrimination of multiple small (< 7 mm) intracranial aneurysms based on machine learning-based cluster analysis

**DOI:** 10.1186/s12883-023-03088-8

**Published:** 2023-01-28

**Authors:** Xin Tong, Xin Feng, Fei Peng, Hao Niu, Xin Zhang, Xifeng Li, Yuanli Zhao, Aihua Liu, Chuanzhi Duan

**Affiliations:** 1 Department of Neurosurgery, Beijing Neurosurgical Institute and Beijing Tiantan Hospital, Capital Medical University, China National Clinical Research Center for Neurological Diseases, 119 Fanyang Road, Beijing, 100070 China; 2 National Key Clinical Specialty, Department of Neurosurgery, Engineering Technology Research Center of Education Ministry of China, Guangdong Provincial Key Laboratory On Brain Function Repair and Regeneration, Neurosurgery Institute, Zhujiang Hospital, Southern Medical University, Guangzhou, China; 3 Department of Neurosurgery, Peking University International Hospital, Beijing, China

**Keywords:** Small multiple intracranial aneurysms, Risk discrimination, Cluster analysis, Machine learning

## Abstract

**Background:**

Small multiple intracranial aneurysms (SMIAs) are known to be more prone to rupture than are single aneurysms. However, specific recommendations for patients with small MIAs are not included in the guidelines of the American Heart Association and American Stroke Association. In this study, we aimed to evaluate the feasibility of machine learning-based cluster analysis for discriminating the risk of rupture of SMIAs.

**Methods:**

This multi-institutional cross-sectional study included 1,427 SMIAs from 660 patients. Hierarchical cluster analysis guided patient classification based on patient-level characteristics. Based on the clusters and morphological features, machine learning models were constructed and compared to screen the optimal model for discriminating aneurysm rupture.

**Results:**

Three clusters with markedly different features were identified. Cluster 1 (*n* = 45) had the highest risk of subarachnoid hemorrhage (SAH) (75.6%) and was characterized by a higher prevalence of familiar IAs. Cluster 2 (*n* = 110) had a moderate risk of SAH (38.2%) and was characterized by the highest rate of SAH history and highest number of vascular risk factors. Cluster 3 (*n* = 505) had a relatively mild risk of SAH (17.6%) and was characterized by a lower prevalence of SAH history and lower number of vascular risk factors. Lasso regression analysis showed that compared with cluster 3, clusters 1 (odds ratio [OR], 7.391; 95% confidence interval [CI], 4.074–13.150) and 2 (OR, 3.014; 95% CI, 1.827–4.970) were at a higher risk of aneurysm rupture. In terms of performance, the area under the curve of the model was 0.828 (95% CI, 0.770–0.833).

**Conclusions:**

An unsupervised machine learning-based algorithm successfully identified three distinct clusters with different SAH risk in patients with SMIAs. Based on the morphological factors and identified clusters, our proposed model has good discrimination ability for SMIA ruptures.

## Background

Previous studies have suggested that the size of an aneurysm is the most important indicator of its risk of rupture in patients with multiple intracranial aneurysms (MIAs) [1]. However, 20–29% of ruptured aneurysms were not the largest in size in patients with MIAs and subarachnoid hemorrhage (SAH) [2, 3]. In patients with MIAs, ruptured aneurysms appear to be smaller (< 7 mm), and smaller aneurysms account for more than half of all ruptured MIAs [2]. Björkman et al. [4] found that of all the ruptured IAs in patients with MIAs, 53.7% and 8.2% were < 7 mm and < 3 mm in size, respectively. The Small Unruptured Intracranial Aneurysm Verification Study in Japan demonstrated that the average annual risk of rupture was 0.34%/year for single unruptured aneurysms ≤ 5 mm in diameter and 0.95%/year for multiple unruptured aneurysms [4]. This suggests that in order to prevent future aneurysmal rupture, MIAs with a diameter of ≥ 4 mm should also be considered for treatment. However, the recently updated guidelines of the American Heart Association and American Stroke Association do not include specific recommendations for patients with small MIAs (< 7 mm) [5]. Therefore, the clinical management of small aneurysms in patients with MIAs should be considered and analyzed, as this can help to improve the prognosis of this group of patients.

Approximately 20–40% of patients with unruptured IAs also harbor additional IAs [6]. Patients with MIAs can share some characteristics with those with single aneurysms; for example, the risk factors for MIAs can be the same as those for aneurysm formation in general. Female sex, age, arterial hypertension, smoking, and familial IA have been found to be the major risk factors for MIA formation [7–9], and they have also been reported to increase the risk of aneurysm rupture [10, 11]. However, compared with single aneurysms, MIAs are generally at a higher risk of growth and rupture [12], suggesting that the development of MIAs is driven by an underlying pathophysiological etiology. The risk factors associated with aneurysm rupture may play a more important role in patients with MIAs than in those with single aneurysm. As such, compared with patients with single aneurysms, those with MIAs are more likely to exhibit natural patterns of grouping (risk factor discrimination), which may be related to the risk of SAH.

In patients with MIAs, the characteristics associated with aneurysm rupture can be divided into patient-level (such as age and sex) and aneurysm-level (such as size and neck width) factors. Each aneurysm in the same patient shares the same patient-level characteristics but can have its own independent aneurysm-level characteristics. Patient-level characteristics can influence the occurrence, growth, and morphology of MIAs, thus influencing aneurysm-level characteristics. Moreover, both patient- and aneurysm-level characteristics can influence aneurysm rupture. As such, some autocorrelation between these factors is inevitable.

In this study, we adopted unsupervised machine learning-based method, cluster analysis, to classify complex epidemiological factors and comorbidities into simple clusters (phenogroupings). Cluster analysis can identify patients with similar clinical characteristics across various groups with different clinical phenotypes (e.g., patients with a higher or lower prevalence of risk factors or comorbidities). Unlike traditional regression analyses, cluster analysis divides a set of data into several distinct categories based on the similarities and differences between the data. Data belonging to the same category have high levels of similarity, whereas those in different categories have low levels of similarity and low levels of cross-category correlation. These phenogroupings are associated with distinct baseline demographic characteristics and comorbidities, highlighting the distinct phenotypes of patients with SMIAs. Management approaches can differ between groups of patients with different clinical characteristics who have MIAs with varying rupture risks. Therefore, in this study, we sought to: (1) analyze patient-level risk factors to identify the clinical phenotypes that are most relevant for discriminating multi-factorial clusters among patients with MIAs; and (2) analyze aneurysm-level risk factors to evaluate the association between these clusters and aneurysm rupture.

## Methods

### Study population

This was a cross-sectional study of consecutive patients with MIAs who attended three medical centers in China (Beijing Tiantan Hospital; Zhujiang Hospital; Peking University International Hospital) between 1 January, 2015, and 1 January, 2019*.* Patients were included if they had at least two saccular and small IAs (< 7 mm). The exclusion criteria were as follows: patients whose largest aneurysm was sized > 7 mm; patients with fusiform or dissecting IAs; patients with arteriovenous malformation/moyamoya disease/arteriovenous fistula; patients with incomplete digital subtraction angiography data or unreadable and unclear 3D rotational angiography.

All aneurysms were divided into the ruptured and unruptured groups based on their presentation at the time of admission. Patients who presented with a suspicion of SAH routinely underwent head computed tomography (CT) and—if the head CT findings were negative—a lumbar puncture. Patients with SAH were included only if the aneurysm responsible for SAH could be determined through microscopic visual assessment or a definitive hemorrhage pattern on CT (localized to one IA).

### Patient-level characteristics

For each patient, data related to their individual characteristics were collected from the medical history recorded by the treating physician during interviews with the patient or their family members. Additional information was collected through a structured questionnaire via telephone interviews.

The individual patient-level characteristics included baseline information and data related to vascular risk factors (Table 1). The following baseline data were collected: age, sex, history of SAH (caused by other aneurysms), family history of IAs (a familial history of aneurysmal SAH and evidence of familial aneurysms [at least 1 first-degree family member with an IA]) [13], and numbers of IAs. The vascular risk factors were as follows: hypertension, hyperlipidemia, cardiovascular disease (angina pectoris, myocardial infarction, or peripheral vascular disease), intracranial atherosclerotic stenosis ≥ 50%, history of stroke (transient ischemic attack or stroke), current smoking (still smoking upon admission); former smoking (used to smoke regularly and quit at least 1 year before admission), and alcohol consumption (current or previous intake of > 5 drinks per day) [14]. In addition, the vascular burden (that is, the number of vascular risk factors) was calculated for each patient [15].

**Table 1:** Characteristics of patients in the three clusters

Patient-level Characteristics	Cluster A	Cluster B	Cluster C	*P*-value
No. (%)	45 (6.8)	110 (16.7)	505 (76.5)	-
Presented with SAH (%)	34 (75.6)	42 (38.2)	89 (17.6)	< 0.001
**Baseline information**				
Age (mean ± SD)	58.5 ± 9.6	56.0 ± 10.8	57.6 ± 10.5	0.264
Female sex (%)	34 (75.6)	28 (25.5)	378 (74.9)	< 0.001
History of SAH (%)	0	32 (29.1)	1 (0.2)	< 0.001
Family history of IAs (%)	45 (100.0)	1 (0.9)	0	< 0.001
Number of IAs (mean ± SD)	2.2 ± 0.4	2.4 ± 0.6	2.4 ± 0.5	0.124
**Vascular risk factors**				
Hypertension (%)	26 (57.8)	79 (71.8)	285 (56.4)	0.012
Diabetes mellitus (%)	9 (20.0)	13 (11.8)	79 (15.6)	0.398
Hyperlipidemia (%)	7 (15.6)	29 (26.4)	96 (19.0)	0.161
Cardiovascular diseases (%)	1 (2.2)	9 (8.2)	60 (11.9)	0.087
History of stroke (%)	8 (17.8)	25 (21.8)	66 (13.1)	0.055
Intracranial atherosclerotic stenosis ≥ 50% (%)	9 (20.0)	38 (35.5)	146 (28.9)	0.185
Current smoking (%)	5 (11.1)	48 (43.6)	86 (17.0)	< 0.001
Former smoking (%)	0	13 (11.8)	12 (2.4)	< 0.001
Alcohol consumption (%)	3 (6.7)	86 (78.2)	8 (1.6)	< 0.001
Number of vascular risk factors (%)				< 0.001
< 3	37 (82.1)	41 (32.0)	416 (82.4)	
≥ 3	8 (17.9)	69 (68.0)	89 (17.6)	

*SAH* Subarachnoid hemorrhage, *IA* Intracranial aneurysm

### Aneurysm-level characteristics

**Fig. 1:**
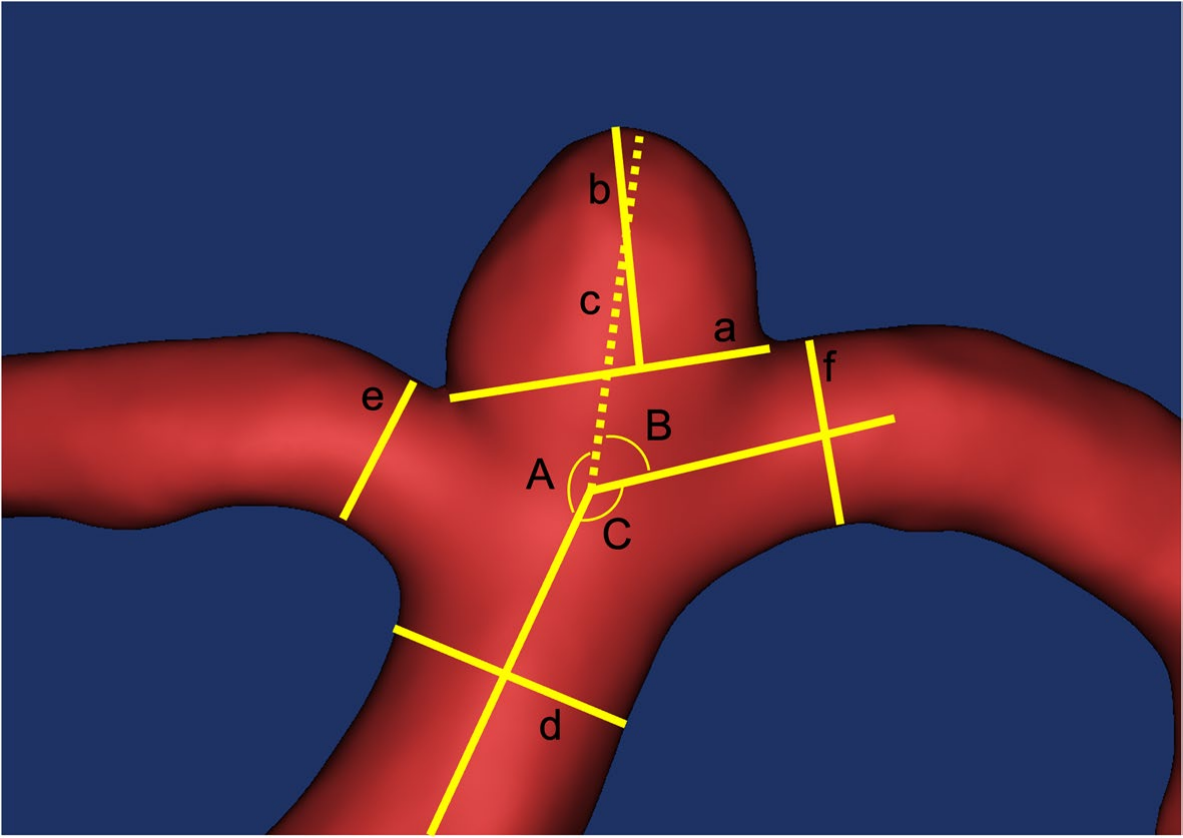
Measurements of the morphological features of aneurysms. a, Neck width; b, height; c, extension of the maximum distance of the dome from the center of the neck plane; d, diameter of the parent artery; e and f, diameter of the branch artery. (A) inflow angle (angle between the parent artery and c); (B) outflow angle; (C) branching angle; aspect ratio, b/a; size ratio, size/d; branching-to-parent ratio, (e + f)/d; neck-to-parent ratio, a/d

The blood vessels were visualized via 3D digital subtraction angiography/CT angiography, and the morphological features and presence of stenosis were determined at the respective center by two experienced readers with more than 10 years of experience. Data recorded from the angiograms included the number, location, and size of IAs; these data will form the basis of a separate study and publication. All 1,427 angiograms were reevaluated and measured at a 0.1-mm scale by two authors (X.T. and X.F.) at the central reading center (Fig. 1). The following parameters were calculated: size of the aneurysm, defined as the maximum distance between any two points on an aneurysmal body; neck width, defined as the maximum distance between any two points on the aneurysmal neck plane; aspect ratio (AR), defined as the ratio of dome height to neck width [16]; size ratio (SR), defined as the ratio of maximum aneurysmal height to the parent diameter; branching-to-parent ratio (BPR; defined as the ratio of the sum of the diameters of branch vessels to the diameter of the proximal main vessel in bifurcation aneurysms; set to 1 for sidewall aneurysms); neck-to-parent ratio (NPR), defined as the ratio of the neck width to the diameter of the parent artery; irregular shape, defined as an aneurysm with multiple lobes, daughter sacs, or other types of wall protrusions [17]; inflow angle, defined as the angle between the parent artery and the direction of the aneurysm [18]; outflow angle, defined as the angle at which the aneurysm flowed outward to the distal part of the artery; main branching angle, defined as the angle of the parent artery (in case of a sidewall aneurysm) or the angle between the parent artery and the daughter branch most approaching 180° (in case of a bifurcation aneurysm) [2] (the sum of the inflow, outflow, and main branching angles was 360°); bifurcation location, defined as aneurysms that had necks located on two vascular branches simultaneously; and posterior circulation location, defined as aneurysms located at the basilar, vertebral, and posterior cerebral arteries.

### Statistical analyses

Continuous variables were compared between groups using the Student's t-test (for normally distributed variables) or Mann–Whitney U test (for non-normally distributed variables). One-way analysis of variance was used to analyze differences in more than two groups. For variables with few missing data, mean imputation was used; 20 variables had no missing data, and 5 had < 10% missing data. Any variable with > 50% missing data was excluded from analysis. Finally, 25 variables were included in the models. The analysis of both datasets—the complete case dataset and imputed dataset—showed similar results.

### Construction of clusters

We used agglomerative hierarchical cluster analysis to classify patients into groups based on 14 patient-level characteristics, including five baseline factors (age, sex, history of SAH, family history of IAs, and number of IAs) and nine vascular risk factors (hypertension, diabetes mellitus, hyperlipidemia, cardiovascular diseases, history of stroke, intracranial atherosclerotic stenosis ≥ 50%, current smoking, former smoking, and alcohol consumption). This is a commonly used method suitable for binary variables [19, 20]. The grouping process was based solely on patient-level data, did not include any aneurysm-level characteristics, and was blinded to the presence of SAH. The algorithm started with individual patients and successively clustered them until the final group contained all patients. The Jaccard similarity coefficient was used as a measure of distance between binary variables and average linkage to define the average distance between data points in separate clusters. All prevalent conditions in this cohort were included in the cluster analysis. The optimum number of clusters was determined using the *NbClust* package in R statistical software. This function provides 30 indices that can be used to determine the optimal number of clusters in a dataset using an objective and data-driven “majority vote” approach [21, 22].

### Identification of morphological determinants for small IA rupture in patients with MIAs

All the included aneurysms were randomly divided into training and testing sets (7:3). Using clusters as a dummy independent variable, multivariable regression models were constructed using logistics, lasso, and ridge regression analyses to assess the risk of aneurysm rupture. These models were named the logistics, lasso, and ridge models, respectively. Receiver operating curves were constructed, and the areas under the curve of these models were compared in order to select the optimal final model. The variates included in the optimal final model were summarized, and the odds ratio (OR) and 95% confidence interval (CI) of each variate were calculated. The variance inflation factor of each variate was used to test collinearity and to identify the morphological determinants for predicting aneurysm rupture. Additionally, the importance of these variables was calculated. In this study, *P* < 0.05 was considered statistically significant, and all calculations were performed on IBM SPSS Statistics for Windows, version 25 (IBM Corp., Armonk, N.Y., USA) and R statistical software.

## Results

### Study population

A total of 660 patients with 1,427 SMIAs (< 7 mm) that met the inclusion and exclusion criteria were included in this study (Table 1). Ruptured IAs were present in 25.0% (165/660) of the included patients and comprised 11.6% (165/1427) of the 1,427 SMIAs. Among the 660 patients, 476 (72.1%) had 2 coexisting IAs, 137 (20.9%) had 3, and 47 (7.1%) had 4 or more coexisting IAs. In terms of size, 378 (26.5%) IAs were < 3 mm, 683 (47.9%) were 3–5 mm, and 366 (25.6%) were 5–7 mm.

### Cluster analysis

**Fig. 2:**
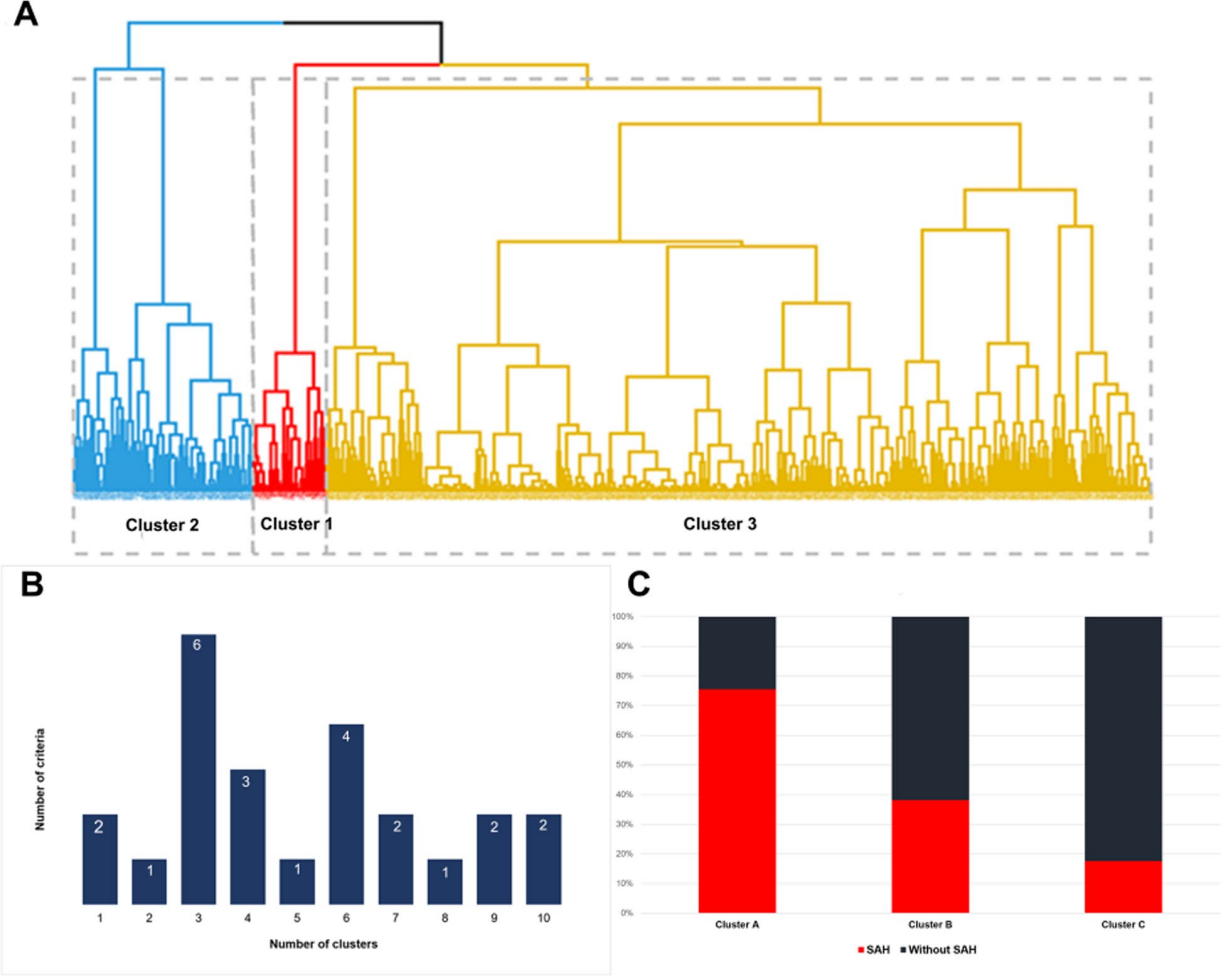
Starting from the bottom, the clusters are progressively joined (at levels of similarity shown at their union) until a single cluster is formed at the top (**A**). NbClust provides the statistically optimum number of clusters, which were three for the index of 6:24 indicators (**B**). Cluster 1 (*n* = 45) shows the highest rate of subarachnoid hemorrhage (SAH) (34/45, 75.6%); Cluster 2 (*n* = 110) shows a moderate risk of SAH (42/110, 38.2%); and cluster 3 (*n* = 505) shows a relatively mild risk of SAH (89/505, 17.6%). Consequently, the cluster variable is significantly associated with the risk of SAH (**C**)

**Fig. 3:**
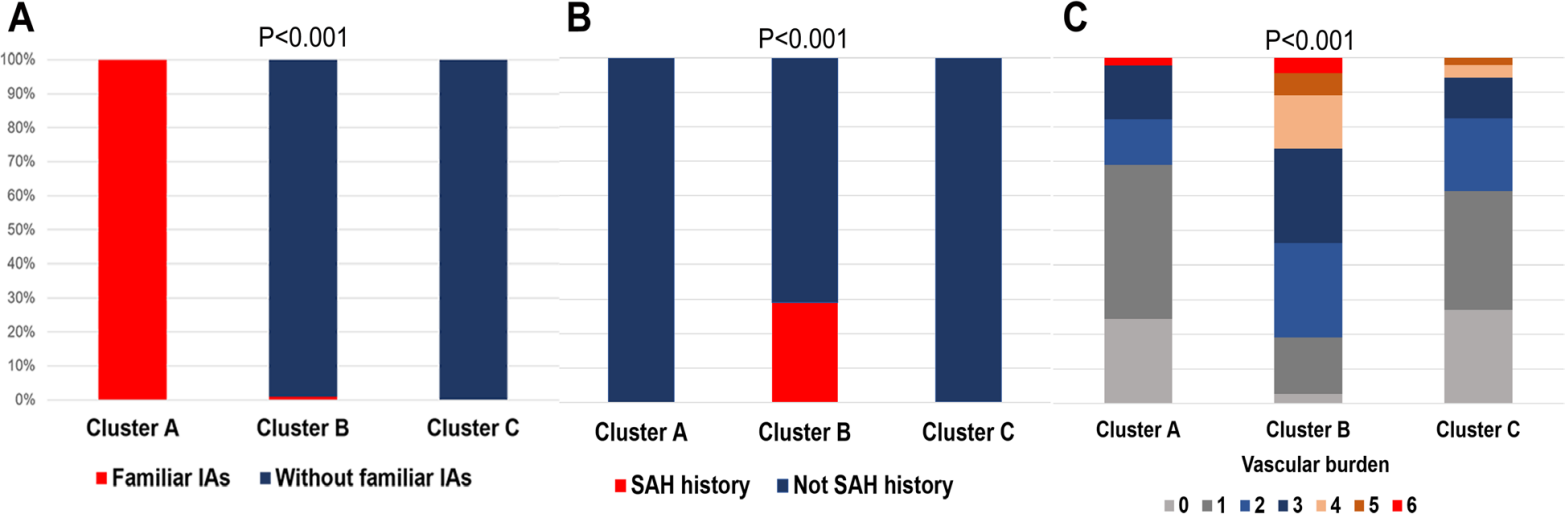
Distribution of patient-related factors in the three clusters. All patients in Cluster 1 (*n* = 45) have a family history of intracranial aneurysms (**A**). Compared with clusters 1 and 3, cluster 2 (*n* = 110) has significantly higher rates of prior aneurysmal subarachnoid hemorrhage due to the rupture of another aneurysm (**B**) and a higher number of vascular risk factors (**C**)

The unsupervised cluster analysis that was blinded to the presence of SAH identified three distinct clusters with different patterns of clinical factors (Fig. 2). The distribution of individual patient-level factors in the three clusters is shown in Table 1 and Fig. 3. There were significant differences in age, sex, number of coexisting IAs, diabetes mellitus, hyperlipidemia, and cardiovascular disease between the clusters (*P* > 0.050). The patients in cluster 1 (*n* = 45) had a family history of IAs (Fig. 3A) and the highest rate of SAH history (34/45, 75.6%, Fig. 2C). Patients in cluster 2 (*n* = 110) had a moderate risk of SAH (42/110, 38.2%) and significantly higher rates of SAH history (29.1%, Fig. 3B), hypertension (71.8%), current and former smoking (55.4%), and alcohol consumption (78.2%). Notably, patients in cluster 2 also had a higher vascular burden (Fig. 3C); 73.6% (81/110) of patients in this cluster had two or more vascular risk factors and 62.7% (69/110) had three or more vascular risk factors. Patients in cluster 3 (*n* = 505) had a relatively mild risk of SAH (89/505, 17.6%) and showed significantly lower rates of SAH history (0.2%), hypertension (56.4%), current and former smoking (19.4%), alcohol consumption (1.6%), and lower vascular burden (only 17.6% of patients had three or more vascular risk factors). Consequently, the clusters were significantly associated with SAH risk (*P* < 0.001, Fig. 2C).

### Associations between patient clusters and SMIA rupture

**Fig. 4:**
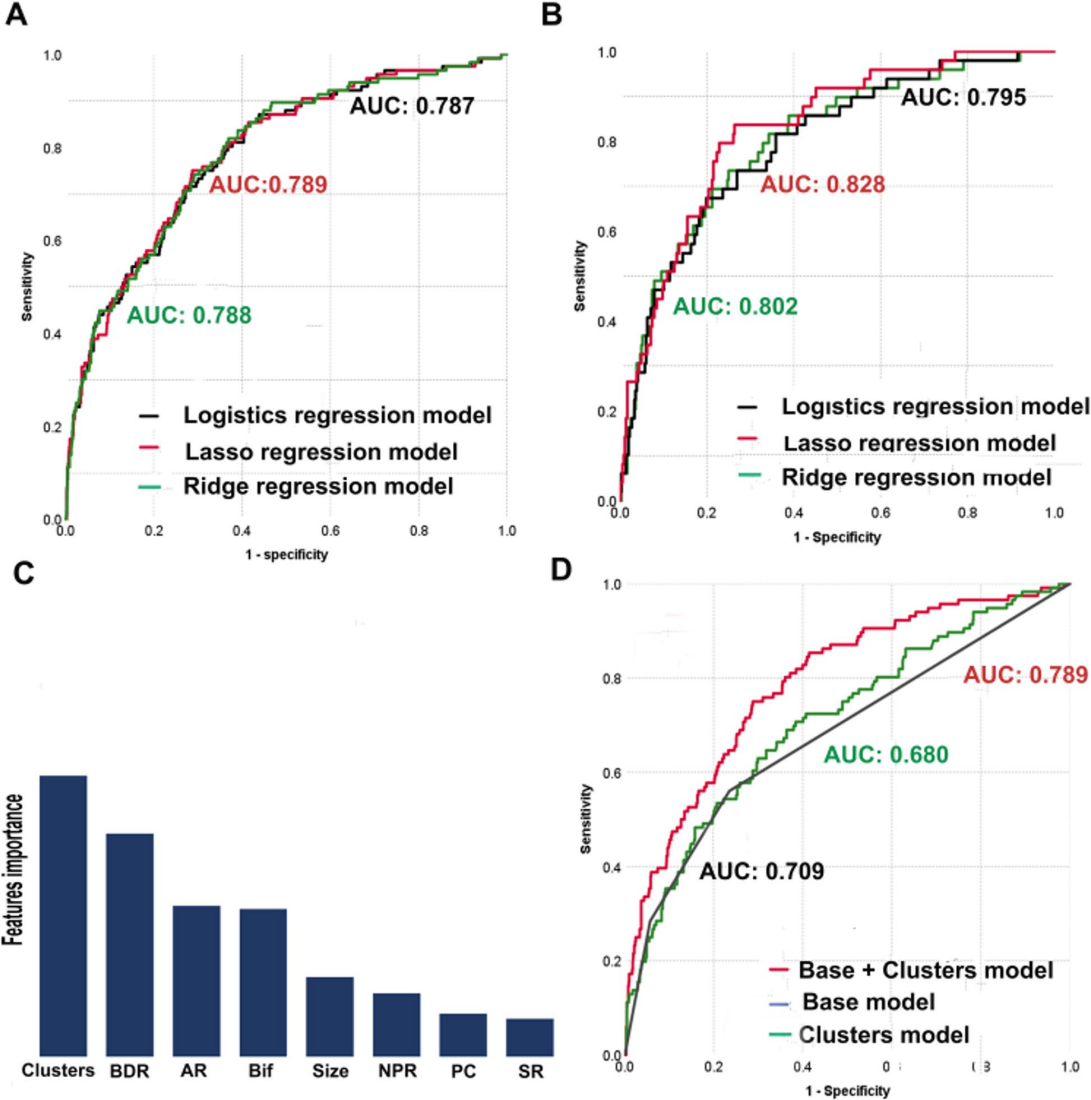
Performance of the logistics, ridge, and lasso regression models combining the cluster variable and aneurysm-level factors in the training (**A**) and test groups (**B**). A comparison of the importance of determinants for rupture discrimination (**C**). A comparison of logistic models based on morphological factors with/without clusters (**D**). BPR, branching-to-parent ratio; AR, aspect ratio; Bif, bifurcation location; NPR, neck-to-parent ratio; PC, posterior circulation; SR, size ratio

The results of cluster analysis and aneurysm-level data were used to verify the performance of various models in the training and test groups (Table 2). The areas under the curve of the logistics, ridge, and lasso models were 0.789 (95% CI, 0.745–0.833), 0.788 (95% CI, 0.742– 0.885), and 0.787 (95% CI, 0.744–0.833) in the training group (Fig. 4A) and 0.828 (95% CI, 0.770–0.833), 0.802 (95% CI, 0.7736–0.868), and 0.795 (95% CI, 0.728–0.861) in the test group, respectively (Fig. 4B). The logistics regression model showed relatively better discrimination performance in both groups and was selected as the optimal model for assessing the risk of SMIA rupture. The areas under the receiver operating characteristic curves were nearly 0.8 for all models, indicating that they showed good discrimination performance overall. Table 3 summarizes the characteristics of the final prediction model. The ORs of the independent variables were as follows: cluster 1 (OR, 7.391; 95% CI, 4.074–13.150; *P* < 0.001), cluster 2 (OR, 3.014; 95% CI, 1.827–4.970; *P* < 0.001), AR (OR, 2.282; 95% CI, 1.409–3.696; *P* = 0.001), bifurcation location (OR, 2.010; 95% CI, 1.190–3.394; *P* = 0.009), NPR (OR, 1.696; 95% CI, 1.027–2.800; *P* = 0.039), size (OR, 1.326; 95% CI, 1.111–1.583; *P* = 0.002), and BPR (OR, 0.335; 95% CI, 0.162–0.693; *P* = 0.003); the variance inflation factors associated with the same variables were 1.86, 1.89, 1.37, 1.22, 1.52, 1.39, and 1.28, respectively.

**Table 2:** Univariate analysis between the unruptured and ruptured groups in the training and test groups

	**Training group (*n* = 1010)**			**Test group (*n* = 417)**		
	**Unruptured**	**Ruptured**	***P*-value**	**Unruptured**	**Ruptured**	***P*-value**
N (%)	894(88.5)	116(11.5)		368(88.2)	49 (11.8)	
Cluster groups (%)			< 0.001			< 0.001
Cluster 1	51(5.7)	33(28.4)		24(6.5%)	14(28.6)	
Cluster 2	159(17.8)	32(27.6)		50(13.6)	12(24.5)	
Cluster 3	684(76.5)	51(44.0)		294(79.9)	23(46.9)	
**Aneurysm-level characteristics**						
Size (mean ± SD)	3.9 ± 1.3	4.7 ± 1.3	< 0.001	4.0 ± 1.4	4.9 ± 1.3	< 0.001
Size Group (%)			0.012			0.006
< 3	291(27.3)	14(13.5)		96(26.1)	4(8.2)	
3–7	573(53.7)	65(62.5)		272(73.9)	45(91.8)	
Neck (mean ± SD)	3.2 ± 1.1	3.5 ± 1.1	0.004	3.3 ± 1.1	3.5 ± 1.2	0.012
AR (mean ± SD)	1.1 ± 0.4	1.3 ± 0.5	< 0.001	1.1 ± 0.4	1.4 ± 0.5	< 0.001
BPR (mean ± SD)	1.1 ± 0.3	1.0 ± 0.4	0.001	1.1 ± 0.3	1.0 ± 0.4	0.023
NPR (mean ± SD)	1.0 ± 0.4	1.1 ± 0.5	0.002	1.0 ± 0.4	1.1 ± 0.4	0.055
SR (mean ± SD)	1.0 ± 0.5	1.4 ± 0.6	< 0.001	1.1 ± 0.5	1.5 ± 0.6	0.004
Location of PC (%)	66(7.4)	13(11.2)	0.149	29(7.9)	7(14.3)	0.134
Irregular shape (%)	259(29.0)	43(37.1)	0.073	89(24.2)	25(51.0)	< 0.001
Bifurcation aneurysm (%)	190(21.3)	37(31.9)	0.010	84(22.8)	17(34.0)	0.083
Inflow angle (mean ± SD)	95 ± 33	103 ± 32	0.008	98 ± 35	110 ± 35	0.022
Outflow angle (mean ± SD)	101 ± 30	103 ± 29	0.612	99 ± 32	102 ± 34	0.538
Main branching angle (mean ± SD)	139 ± 33	134 ± 33	0.086	137 ± 34	131 ± 37	0.239

*SAH* Subarachnoid hemorrhage, *AR* Aspect ratio, *BPR* Branching-to-parent ratio, *NPR* Neck-to-parent ratio, *PC* Posterior circulation

**Table 3:** Multivariate analysis of the characteristics of 1427 small (< 7 mm) intracranial aneurysms in 660 patients

	**OR**	**95% CI**	***P*-value**	**VIF**
Bifurcation location	2.010	1.190–3.394	0.009	1.217
BPR	0.335	0.162–0.693	0.003	1.283
Size	1.326	1.111–1.583	0.002	1.388
AR	2.282	1.409–3.696	0.001	1.371
NPR	1.696	1.027–2.800	0.039	1.517
Clusters				
Cluster 3	Reference	Reference	Reference	
Cluster 1	7.391	4.074–13.150	< 0.001	1.860
Cluster 2	3.014	1.827–4.970	< 0.001	1.891

*OR* Odds ratio, *BPR* Branching-to-parent ratio, *AR* Aspect ratio, *NPR* Neck-to-parent ratio, *VIF* Variance inflation factor

The size cutoff for a higher risk of aneurysm rupture was 4.6 mm, as determined by the Youden Index. Notably, the variable importance of clusters was highest in the lasso model (relevance from high to low: clusters, BPR, AR, bifurcation, size, NPR, posterior circulation, and size ratio; (Fig. 4C) and third highest in the ridge model (relevance from high to low: AR, irregular shape, clusters, bifurcation, size ratio, posterior circulation, neck, BPR, and number of coexisting aneurysms). These results indicate that the cluster variable can be a vital independent risk factor for assessing the risk of SMIA rupture.

### Incremental predictive value of clusters for outcome prediction

The baseline regression model for SMIAs was constructed using the individual morphological features of aneurysms. The addition of the cluster variable to the base model significantly improved its integrated discrimination ability, resulting in accurate reclassification of the variables in terms of SMIA rupture (C statistics: 0.709 vs. 0.789; Fig. 4D).

## Discussion

This study revealed three main findings. First, unsupervised cluster analysis successfully identified three specific groups in a large cohort of patients with small (< 7 mm) MIAs who were referred for the evaluation of SAH risk. The patient groups were mainly characterized by differences in a family history of IAs, SAH history, hypertension, smoking status, and alcohol consumption, corresponding to differences in SAH rates. Second, the cluster variable was found to be a critical independent risk factor for evaluating the risk of individual aneurysms, and this result was replicated in the validation group. Third, our machine learning-based model, which combined the cluster variable with individual morphological factors, showed good discrimination ability for SMIA rupture.

One of the most advantageous features of machine learning algorithms is that they can discover hidden patterns in heterogeneous data. Moreover, cluster analysis can access complex nonlinear interactions and analyze the intrinsic structure of data [19]. Unsupervised machine learning algorithms have previously been used to determine the size cutoff for the IA population based on morphological and hemodynamic features [20]. To the best of our knowledge, this study is the first to analyze clinical factors using an unsupervised machine learning algorithm to successfully group patients with SMIAs according to their risk of SAH.

The phenogroups derived from cluster analysis demonstrated varying levels of risk for SAH, ranging from low (cluster 3) to high (cluster 1). Cluster 1 was associated with the highest rate of SAH. All patients in cluster 1 had a family history of IA, which is recognized as an important risk factor for aneurysm formation, change, and rupture (according to the updated guidelines of the American Heart Association and American Stroke Association) [23]. Cluster 2 was associated with a moderate risk of SAH (cluster 2); patients in this groups showed the highest levels of current and former smoking [24], previous history of SAH [10], hypertension [25], and alcohol consumption [26]. Moreover, cluster 2 had more patients with three or more vascular risk factors, suggesting that a high vascular burden may be an important and novel indicator for evaluating the risk of aneurysm rupture [15]. These results showed that patients with MIAs who have a family history of IA or/and a higher vascular burden should be managed with more caution in the clinical setting. MIAs and a family history of IA could be related due to underlying genetics or common environmental exposure/lifestyle, and few studies have investigated whether these parameters could be common risk factors for SHA.

The phenogroups facilitated our assessment of the risk of aneurysm rupture. As expected, when multivariate analyses were performed using the phenogroups as a dummy variable, all three machine learning models consistently showed that phenogroups were significantly associated with the rupture risk of individual SMIAs. The cluster with the highest risk of SHA had the highest OR in the logistics model, followed by the clusters with medium (cluster 2) and low (cluster 3) risk. Moreover, the clusters were the most important determinant of aneurysm rupture, followed by BPR, AR, bifurcation, and size (Fig. 4C). We found that the addition of the cluster variable to the model greatly improved its discrimination ability, leading to the reclassification of individual SMIAs (Fig. 4D). Our study proves that an unsupervised clustering method that segregates patients into distinct phenogroups with distinct risks of aneurysm rupture may present a novel tool for risk assessment among patients with SMIAs. The clinical applications of cluster-based approaches may also be enhanced by incorporating a broader range of data, including the findings on high-resolution magnetic resonance imaging and hemodynamic measurements. Nevertheless, these hypotheses need to be tested in future studies.

In a previous study, aneurysm size was ranked as the most important risk factor for aneurysm rupture [5]. Moreover, larger aneurysms are widely accepted to be more dangerous than smaller ones. A systematic review of the growth and rupture risk of ≤ 7 mm IAs concluded that 12 out of 13 studies reported a rupture rate of < 1%, whereas 1 reported a rupture rate of 3.10% [27]. The recently updated guidelines of the American Heart Association and American Stroke Association [23] for managing unruptured IAs do not include specific recommendations for treating aneurysms ≤ 7 mm. However, there is a controversy regarding recommendations for patients with MIAs, as ruptured MIAs are often small in size [27, 28]. In our study, 25.0% (165/660) of patients with SMIAs sized ≤ 7 mm experienced aneurysm rupture, which is consistent with the findings of previous studies [27, 28]. The final lasso and ridge models found that in multiple aneurysms sized ≤ 7 mm, size was also an important independent risk factor aneurysm. Further analysis revealed that the size cutoff determined by the Youden Index for evaluating a higher risk of aneurysm rupture was 4.6 mm. This finding was similar to that of the Small Unruptured Intracranial Aneurysm Verification Study, which suggested that MIAs sized ≥ 4 mm should be considered for treatment in order to prevent future aneurysmal rupture [28].

Aneurysm rupture is significantly associated with various risk factors, including bifurcation aneurysm [27, 29, 30] and AR [31, 32]. In this study, we used two novel morphological parameters in our analyses: BPR and NPR. Multivariate analysis showed that a smaller BPR was associated with a greater risk of aneurysm rupture. This may be due to the smaller diameter of the distal branch vessel, which renders it more susceptible to large blood flow velocity, thereby increasing the risk of rupture [33, 34]. Although wide-necked aneurysms have been defined as those with a neck width of > 4 mm, this criterion may be too absolute [35]. Blood flow through aneurysms of different-sized parent arteries may be different, even when the aneurysms have similar neck widths [35]. NPR, also known as the “neck ratio,” is a novel index defined as the ratio of the neck width of a clinical aneurysm to the diameter of the parent artery. NPR is associated with the incomplete occlusion of flow diverter-treated sidewall aneurysms [36, 37], and we found that a larger NPR was associated with a greater risk of aneurysmal rupture. This may be because a larger neck width entails greater blood flow, leading to greater shear stress on the vessel wall [38].

The final model did not reveal any significant associations between size ratio, posterior circulation aneurysm, irregular shape, in/outflow angle, branching angle, and aneurysm rupture. Nevertheless, our findings do not diminish the importance of these predictive factors for MIA rupture. Instead, we suggest that these are not significantly associated with aneurysmal rupture after adjusting for the other predictors included in our models. In fact, size ratio and posterior circulation were included in the lasso model for discriminating the risk of rupture (Fig. 4C).

### Limitations

This study has some limitations. First, the aneurysms in this study were selected from a multi-center cross-sectional database; therefore, the generalizability of our proposed model may not be absolute. Further, we cannot address whether this model can predict impending rupture in unruptured IAs. Second, we lack data on other potentially relevant characteristics such as genetics, blood pressure, hemodynamic parameters, and vascular wall enhancement (as visualized on magnetic resonance imaging). It is plausible that these factors may lead to phenotypic clustering patterns that are distinct from those observed in our study. Finally, we did not include small aneurysms in patients with multiple aneurysms > 7 mm, which may cause a selection bias. These patients were excluded under the assumption that the risk of SAH would be affected by the presence of a larger aneurysm. Further prospective cohort studies must be conducted to address this issue.

## Conclusions

In conclusion, we used an unsupervised clustering algorithm to analyze the clinical data of a large cohort of patients with SMIAs (< 7 mm). Based on the results, we identified three specific groups with significantly different risks of SAH. Our findings suggest that the cluster variable can be a critical independent risk factor for individual aneurysm rupture, and this result was replicated in the validation group. Our machine learning models combine cluster analysis with individual morphological factors and show very good discrimination ability for the risk of rupture of individual SMIAs.

## Data Availability

The datasets used and analyzed during the current study are available from the corresponding author on reasonable request.

## Abbreviations

AR: Aspect ratio
BPR: Branching-to-parent ratio
IA: Intracranial aneurysms
MIA: Multiple intracranial aneurysms
NPR: Neck-to-parent ratio
SAH: Subarachnoid hemorrhage
SMIA: Small multiple intracranial aneurysms
PC: Posterior circulation
VIF: Variance inflation factor
